# Increased Antibacterial and Antibiofilm Properties of Silver Nanoparticles Using Silver Fluoride as Precursor

**DOI:** 10.3390/molecules25153494

**Published:** 2020-07-31

**Authors:** Federico Bertoglio, Lorenzo De Vita, Agnese D’Agostino, Yuri Diaz Fernandez, Andrea Falqui, Alberto Casu, Daniele Merli, Chiara Milanese, Silvia Rossi, Angelo Taglietti, Livia Visai, Piersandro Pallavicini

**Affiliations:** 1 Dipartimento di Medicina Molecolare, Centro per le Tecnologie della Salute (Center for Health Technologies – CHT), UdR INSTM, Università di Pavia, viale Taramelli 3/b, 27100 Pavia, Italy; livia.visai@unipv.it; 2 Scuola Universitaria Superiore IUSS Pavia, Palazzo del Broletto-Piazza della Vittoria 15, 27100 Pavia, Italy; 3 Dipartimento di Chimica, Università di Pavia, viale Taramelli 12, 27100 Pavia, Italy; lorenzo.devita01@universitadipavia.it (L.D.V.); agnese.dagostin@polimi.it (A.D.); daniele.merli@unipv.it (D.M.); chiara.milanese@unipv.it (C.M.); angelo.taglietti@unipv.it (A.T.); 4 Department of Chemistry, University of Liverpool, Liverpool L69 3BX, UK; Yuri.Diaz-Fernandez@liverpool.ac.uk; 5 NABLA Lab, Biological and Environmental Sciences and Engineering (BESE) Division, King Abdullah University of Science and Technology (KAUST), Thuwal 23955-6900, Saudi Arabia; andrea.falqui@kaust.edu.sa (A.F.); alberto.casu@kaust.edu.sa (A.C.); 6 Dipartimento di Scienze del Farmaco, Università di Pavia, viale Taramelli 12, 27100 Pavia, Italy; silvia.rossi@unipv.it; 7 Dipartimento di Medicina Occupazionale, Tossicologia e Rischi Ambientali, Istituti Clinici Scientifici Maugeri S.p.A Società Benefit, IRCCS, Via S. Boezio 28, 27100 Pavia, Italy

**Keywords:** silver nanoparticles, fluoride, antibacterial, biofilm, antibiofilm, ionic silver

## Abstract

Silver nanoparticles were produced with AgF as the starting Ag(I) salt, with pectin as the reductant and protecting agent. While the obtained nanoparticles (pAgNP-F) have the same dimensional and physicochemical properties as those already described by us and obtained from AgNO_3_ and pectin (pAgNP-N), the silver nanoparticles from AgF display an increased antibacterial activity against *E. coli* PHL628 and *Staphylococcus epidermidis RP62A* (*S. epidermidis* RP62A), both as planktonic strains and as their biofilms with respect to pAgNP-N. In particular, a comparison of the antimicrobial and antibiofilm action of pAgNP-F has been carried out with pAgNP-N, pAgNP-N and added NaF, pure AgNO_3_, pure AgF, AgNO_3_ and added NaF and pure NaNO_3_ and NaF salts. By also measuring the concentration of the Ag^+^ cation released by pAgNP-F and pAgNP-N, we were able to unravel the separate contributions of each potential antibacterial agent, observing an evident synergy between p-AgNP and the F^−^ anion: the F^−^ anion increases the antibacterial power of the p-AgNP solutions even when F^−^ is just 10 µM, a concentration at which F^−^ alone (i.e., as its Na^+^ salt) is completely ineffective.

## 1. Introduction

Silver nanoparticles (AgNP) are among the most popular nanomaterials, returning more than 73,000 results in a topic search on Web of Science [1]. Among these results, ~25% are in regards to the antimicrobial use of AgNP. In turn, the antibacterial use of AgNP represents, in the scientific literature, the largest subset of long known antibacterial properties of silver in any of its forms, including bulk metal and Ag^+^ salts and complexes [2]. Although many synthetic approaches exist, the most common synthesis of AgNP exploits the straightforward reduction of Ag(I) in water (E°Ag^+^/Ag = + 0.800 V) with NaBH_4_. The starting material is invariably AgNO_3_. In our ten years of experience in the field, we have perused hundreds of papers describing AgNP prepared by Ag^+^ reduction in water, and we have never read of a precursor differing from AgNO_3_. Besides our personal memories, a reliable indication of such a trend comes from the highly cited Chernousova and Epple 2013 review on silver as antibacterial [3]: we have checked all the referenced papers that make use of AgNP, finding only syntheses starting from AgNO_3_ with NaBH_4_ as the reductant. The latter yields borates as the BH_4_^−^ oxidation product. Nitrate and borates typically remain in the AgNP colloidal solutions used for biomedical studies and their toxicity [4,5] is simply neglected. In this regard, hundreds of papers are now surfacing stating that while still using AgNO_3_ to obtain AgNP, we can at least avoid the potential toxicity of the BH_4_^−^ reaction products. In such papers, natural, allegedly biocompatible reductants are used, such as proteins, polysaccharides, alkaloids, enzymes, and quinones, typically from plant extracts [6,7,8,9]. In this context, we recently published a synthesis of AgNP with the natural polysaccharide pectin, starting from AgNO_3_ [10]. The obtained AgNP are stable for months, they release only traces of Ag^+^ with time (1 ppm range after weeks) and exert an extraordinarily efficient antibacterial and antibiofilm action, thanks to the combined effect of Ag^+^ traces and of the direct AgNP-bacteria contact, [10,11,12,13] amplified by the delivery-promoting ability of pectin adhering to the bacterial membranes [14,15].

The main, simple reason for extensive use of the AgNO_3_ in AgNP synthesis is that silver salts are generally very poorly or negligibly soluble, while silver nitrate has a solubility as high as 122 g/100 g H_2_O at 0 °C [16]. However, differing from other insoluble halogenides, AgF has a solubility even larger than AgNO_3_, 182 g/100 g H_2_O at 0 °C [16]. Interestingly, the F^−^ anion is used worldwide in caries prophylaxis. Although its action mechanism is not yet fully explained, a major role seems to be the promotion of tooth remineralization and the reduced acid production from glucose due to the F^−^ weak base nature [17] (pK_b_ F^−^ = 10.86). Beside this, F^−^ inhibition of enzymes involved in the metabolism of bacteria in caries biofilm has also been demonstrated [18,19,20]. Moreover, it has been reported that F^−^ exerts an adverse effect, although extremely weak, against one of the reference strains for antimicrobial studies, *Escherichia coli* (*E. coli*): an MIC (minimum inhibitory concentration) as high as 0.200 M (8.4 g/L) was reported for NaF [21]. The joint advantageous properties of Ag^+^ and F^−^ have been exploited in the extensive use of the silver diamine complex [Ag(NH_3_)_2_]F as anti-caries [22]. In more recent years, solutions of silver nanoparticles obtained from AgNO_3_ and NaBH_4_ were implemented with huge NaF quantities (~0.5 M concentration, corresponding to ~22 g/L), observing for this mixture a strong antimicrobial effect against *Streptococcus mutans*, comparable to that of [Ag(NH_3_)_2_]F [23], and excellent anticaries properties [24]. For the present paper, we used AgF as the starting material to obtain AgNP in pectin; namely, pAgNP-F. The chemical-physical and optical characterization of pAgNP-F demonstrate that they are identical to AgNP obtained in pectin from AgNO_3_ (pAgNP-N). However, we measured the pAgNP-F antibacterial activity against *E. coli* PHL628 and *Staphylococcus epidermidis RP62A* (*S. epidermidis* RP62A), both as planktonic strains and as their biofilms, and found significant differences with respect to pAgNP-N. In particular, a comparison of the antimicrobial and antibiofilm action of pAgNP-F has been carried out with pAgNP-N, pAgNP-N and added NaF, pure AgNO_3_, pure AgF, AgNO_3_ and added NaF and pure NaNO_3_ and NaF salts. By also measuring the concentration of the Ag^+^ cation released by pAgNP-F and pAgNP-N, we were able to unravel the separate contributions of each potential antibacterial agent, observing an evident synergy between p-AgNP and the F^−^ anion: the F^−^ anion increases the antibacterial power of the p-AgNP solutions even when F^−^ is just 10 µM, a concentration at which F^−^ alone (i.e., as its Na^+^ salt) is completely ineffective.

## 2. Results and Discussion

### 2.1. Preparation and Properties of pAgNP-F 

**Figure 1:**
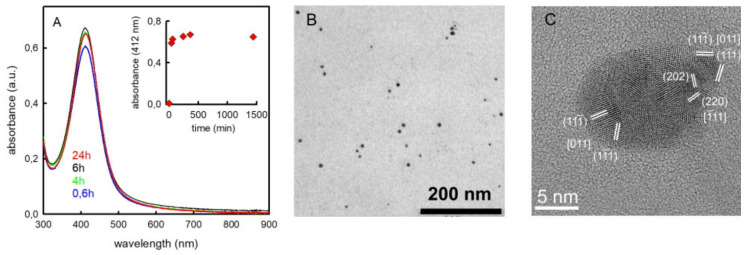
pAgNP-F sample. (**A**): absorbance spectrum measured in the 300–900 nm range. The inset shows the absorbance measured at a wavelength of 412 nm over time (time range: 1500 min). (**B**): conventional transmission electron microscopy (TEM) showing nanoparticles with spheroidal shapes. (**C**): High-resolution transmission electron microscopy (HRTEM) image of a single, representative polycrystalline nanoparticle, showing the {220} and {111} lattice planes of Ag, corresponding to interplanar spacings of 1.44 Å and 2.36 Å. The combinations of interplanar spacings and angular relationships are in accordance with the formation of polycrystalline nanoparticles.

We adopted the same conditions that were optimal in the pAgNP-N synthesis [10], i.e., a 1.0% *w/w* solution of pectin from citrus in water. Pectin acts both as a reductant for Ag^+^ and as a stabilizer for AgNP. AgF was added at 0.001 M concentration; pH was regulated to basic values (10.5) by addition of 0.5 M NaOH up to a 0.025 M concentration. Ag^+^ reduction started only at pH >> 7, as it is due to the oxidation of the –(OH)C–C(OH)– groups to aldehydes in the d-galacturonic acid chain of pectin, a reaction that requires a basic environment to proceed [10]. We carried out syntheses either at 25 °C or at 60 °C, reaching completion after 24 h and 4 h, respectively, as seen by the spectrophotometric analysis of the reaction mixture that follows the LSPR absorption of the spherical pAgNP-F (λ_max_ = 412 nm). Figure 1A displays the series of spectra obtained at 60 °C and (inset) the absorbance at 412 nm vs. time. The pH values at the end of the synthesis were in the 10.7–10.9 range at both temperatures.

The pAgNP-F products obtained at the two temperatures have a spherical shape and identical dimensions within standard deviation. The average d is 7.0 nm (σ = 1.9 nm) and 7.2 nm (σ = 2.7 nm) for 25 and 60 °C preparations; see Figure 1B for a transmission electron microscopy (TEM) image in the latter case (spectra vs time and TEM images for 25 °C preparations are included in Supplementary Materials SM1). High Resolution TEM (HRTEM) imaging was also performed, showing prevalently polycrystalline nanoparticles with an approximately spherical shape; see Figure 1C. HRTEM imaging carried out on pAgNP-N (Supplementary Materials SM1) found no significant difference in the nanoparticles shape and crystallinity with respect to pAgNP-F, thus indicating that the enhanced antibacterial properties observed for the pAgNP-F (vide infra) are not related to size, shape and structure inequalities with respect to pAgNP-N. Also, reaction times to reach pAgNP-F growth completion and their LSPR absorptions are identical to those observed for pAgNP-N. All data agree that F^−^ and NO_3_^−^ counter anions do not play a relevant role in the reduction process of the relative Ag^+^ salts to nanoparticles. Coherently, this could be expected on the basis of the high solubility of the two salts and on the negligible coordination ability towards Ag^+^ of both F^−^ and NO_3_^−^. A fundamental parameter to take into account for antibacterial experiments is the residual free Ag^+^ after reduction in the pAgNP-F synthesis. We measured its concentration using the same potentiometric titration method established for pAgNP-N [25], finding only 0.78 ppm (25 °C, 24 h) and 0.75 ppm (60 °C, 4 h) of free Ag^+^ at the end of the synthesis, which corresponds to 0.1% of the total Ag, and once again such values coincide with those found for pAgNP-N, for which 0.79 ppm of free Ag^+^ were found for 60 °C (4 h) preparations [10]. The synthesis carried out at 60 °C for 4 h was adopted as a standard and used for all the following studies. Further characterization on these pAgNP-F included FT-IR, carried out on solid samples and isolated by precipitation after 1:10 dilution in ethanol, followed by ultracentrifugation and vacuum desiccator drying (Supplementary Materials SM2). Comparison with the FT-IR spectrum of pure unreacted pectin shows the disappearance of the band at 1735 cm^−1^, due to the –COOH and COOCH_3_ carbonyl stretching, as in the basic conditions adopted for the synthesis carboxylic acid deprotonate and esters hydrolyze to carboxylates. Coherently, the –COO^−^ band at 1608 cm^−1^ strongly increases in pAgNP-F with respect to pure pectin. Thermogravimetric analysis run on solid samples isolated with the same procedure, after a first H_2_O loss at lower temperatures, shows a main weight loss (34%) at 200 °C, attributable to pectin decomposition (Supplementary Materials SM3). A higher temperature mass loss was also observed (Supplementary Materials SM3), and regards further processes of the decomposed pectin matrix, as it is observed also in TGA carried out on pure pectin [10]. Samples of pAgNP-N were reprepared for comparing bacteria treatments in this research. All the characterizations were also run on these fresh samples for comparison. The data listed in Table 1 further confirm that pAgNP-F and pAgNP-N are indistinguishable on the chemical-physical point of view. Finally, it must be stressed that only 1/100 of the pectin galacturonic acid units react with Ag^+^ in the used synthetic conditions [10]. All of this allows us to state that the pAgNP-F employed for the following microbiological experiments are aqueous solutions containing ~1% (10 g/L) of hydrolyzed pectin, 0.107 g/L of colloidal silver (0.001 mol Ag/L), 0.0189 g/L F^−^ (0.001 mol/L) and residual excess NaOH leading to pH 10.70–10.90. The pAgNP-N solutions are identical, except that NO_3_^−^ is present (0.0620 g/L, 0.001 mol/L) instead of F^−^.

### 2.2. Antimicrobial Activity on Planktonic Bacteria

**Figure 2:**
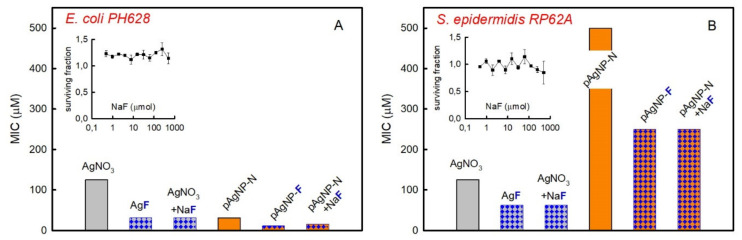
Minimum inhibitory concentration (MIC) values for treatments on planktonic bacteria. (**A**): *E. coli PH628*, grey bars refer to the effect of ionic silver and orange bars to the effect of pAgNP, blue checkers visually evidence data for treatments containing F^−^; (**B**): same, for *S. epidermidis* RP62A. Insets: surviving fraction vs micromolar concentration of NaF on treatment with the pure sodium salt for *E. coli PH628* (**A**) and *S. epidermidis RP62A* (**B**).

The antimicrobial activity of pAgNP-F and pAgNP-N was measured on planktonic *E. coli* PH628 and *S. epidermidis* RP62A, which themselves are biofilm-forming Gram negative and Gram positive bacterial strains, respectively. We used the prepared solutions assuming a total Ag(0) content of 1000 µM, given the negligible quantity of free Ag^+^. We followed the standard geometric dilution method (see Materials and Methods 4.4) to calculate the MIC (minimum inhibitory concentration) values, expressed as total Ag concentration. Despite the basicity of the pAgNP-F and pAgNP-N solutions (Table 1), the pH of all the medium-diluted solutions used on bacterial strains is ~7.4, thanks to the buffered culture medium. The results are visually shown in Figure 2A (*E. coli*) and 2B (*S. epidermidis*), orange background columns. The MIC found for pAgNP-F was 11.7 µM for *E. coli* and 250 µM for *S. epidermidis*. The MIC found for pAgNP-N was instead 31.25 µM and 500 µM for *E. coli* and *S. epidermidis*, respectively, the latter result replicating what we have found using pAgNP-N on the same strains in our previous paper [10]. The much higher MIC value found for both AgNP preparations for *S. epidermidis* with respect to *E. coli* is not surprising and is correctly inscribed in the established higher resistance of the Gram positive bacteria to Ag(0), attributed to their thicker peptidoglycan cell wall [11,26,27]. On the other hand, the lower MIC found for pAgNP-F with respect to pAgNP-N with both *E. coli* and *S. epidermidis* is an unexpected result. In the previous section we showed that pAgNP-F and pAgNP-N are identical on the dimensional and crystalline point of view. Their solutions, used to treat bacteria, have identical total Ag concentration and free Ag^+^ values are also identical within the experimental error. They differ only for the concentration of the anion coming from the starting salt used in the synthesis: pAgNP-N solutions contain 1000 µM NO_3_^−^, pAgNP-F solutions contain 1000 µM F^−^. It has to be stressed that in the standard geometric dilution method, the F^−^ and NO_3_^−^ concentrations undergo the same step decrease as Ag. It must be concluded that it is the F^−^ anion that makes pAgNP-F a stronger antimicrobial than pAgNP-N. This is supported by the MIC of bacterial strains treated with solutions of pAgNP-N to which 1000 µM NaF was added after synthesis: in this case, an identical MIC value was found with respect to pAgNP-F with both strains. 

For sake of comparison, we also determined the MIC for Ag^+^ solutions using either nitrate or fluoride silver salts, and as a further control equimolar quantities of AgNO_3_ and NaF (Figure 2, grey background columns). MIC on *E. coli* are 125 µM for AgNO_3_ and 31.25 for AgF, that is a noticeably lower value. It is also interesting to note that silver nanoparticles in the presence of F^−^ are even a better antimicrobial than AgF. An MIC value identical to that of AgF is found using solutions of AgNO_3_ with NaF added at the same concentration. 

A similar picture is found for *S. epidermidis*, Figure 2B, as ionic silver gives lower MIC values when F^−^ is the counter anion or NaF is added to AgNO_3_. On the other hand, the higher MIC values found on *S. epidermidis* for pAgNP-N and pAgNP-F with respect to ionic silver are coherent with the higher resistance of Gram positive bacteria to the nanomechanical action of silver nanoparticles [11] and to silver nanoparticles uptake [13].

Remembering that an MIC as high as 0.200 M (2 × 10^5^ µM) was reported for pure NaF on *E. coli* [21], we also checked any possible intrinsic antibacterial effects of the sole fluoride anion in the concentration range used in this paper, that is 100–1000 fold lower. NaF solutions displayed no effect on the viability of *E. coli* up to 500 µM (inset in Figure 2A) and a small decrease in the surviving fraction of *S. epidermidis* (0.85) was observed only when AgF concentration was at its highest, i.e., 500 µM, inset in Figure 2B. Pectin alone had no effect on both bacteria viability (results not shown).

### 2.3. Antimicrobial Activity on Biofilms

**Figure 3:**
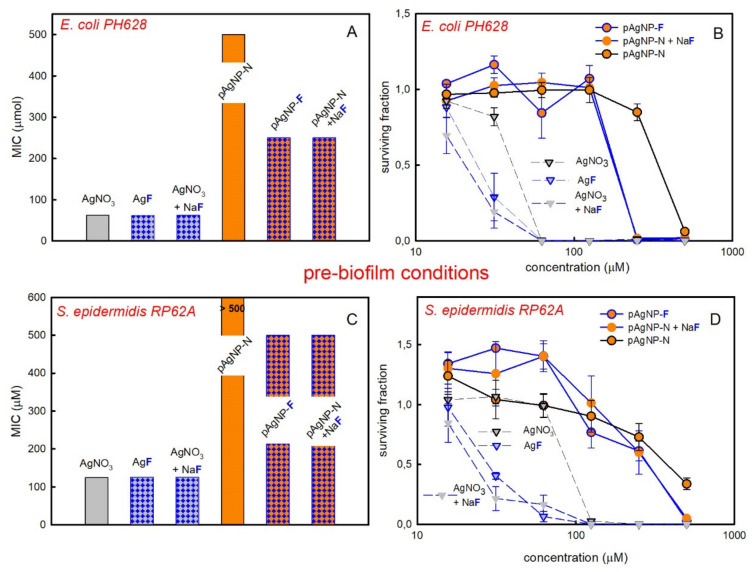
Data for pre-biofilm conditions. (**A**): MIC values for *E. coli PH628*, grey bars refer to the effect of ionic silver and orange bars to the effect of pAgNP, blue checkers visually evidence data for treatments containing F^−^. (**B**): surviving fraction values for *E. coli PH628* vs silver concentration; orange circles refer p-AgNP, blue outlines and/or blue solid lines (drawn to guide the eye) define treatments containing F^−^ (see legend in figure for exact match); (**C**): same as (**A**), for *S. epidermidis RP62A*; (**D**): same as (**B**), for *S. epidermidis RP62A*.

The *E. coli* PH628 and *S. epidermidis* RP62A strains are biofilm producers. We evaluated their viability in biofilm-forming conditions (0.25% Glucose, Glc, supplement in the medium) when treated with the same series of silver nanoparticles and salts used for planktonic conditions. Two setups were used: (i) Pre-biofilm formation, with the bacteria plated in the presence of the investigated agent and Glc in the medium; ii) Post-biofilm formation, with the biofilm allowed to form, and, after that, carrying out addition of the chosen agent. In all cases, the exposure time was 24 h. Results are shown in Figure 3 for both strains in pre-biofilm conditions. The effect of treatments with nanoparticles is always lower with respect to what found with silver salts and as a consequence lower MIC values are found than in the case of ionic silver, see Figure 3A,C. It has been hypothesized that this is due the easier cellular uptake of silver ions in such conditions, thanks to the formation of Ag^+^-glucose complexes [28,29] Moreover, also a decreased mobility of nanoparticles in biofilms with respect to ions has to be taken into account [30]. This is due to their different dimensions and to the trapping effect exerted by EPS (Extracellular Polymeric Substance) on nanoparticles.

Besides this, when pAgNP-F are used instead of pAgNP-N, an increased antibiofilm action, i.e., lower MIC values, were found for both strains. In the case of *S. epidermidis* and pAgNP-N a surviving fraction value of 0 was not reached even at the maximum used concentration (MIC > 500 µM, not determined), although a decrease of the bacterial surviving fraction is clearly observed at higher silver concentrations in Figure 3D. Differently, pAgNP-F were able to exert a complete inhibition of bacterial proliferation at 500 µM. With *E. coli*, the MIC for pAgNP-N is 500 µM, while with pAgNP-F it drops to 250 µM. Noticeably, this is due again to the combined action of pAgNP and of the F^−^ anion, as the MIC obtained using solutions of pAgNP-N with NaF added post synthesis at a 1000 µM concentration are identical to those of pAgNP-F for both strains. The examination of the effect of Ag^+^ ions in pre-biofilm conditions brings further interesting results. AgF has an identical MIC value as AgNO_3_ on *E. coli*, 62.5 µM, and the same MIC is also found for solutions of AgNO_3_ + NaF, Figure 3A. However, observing the trend of the bacterial surviving fractions in Figure 3B, it can be seen that while at 31.25 µM AgNO_3_ the surviving fraction of *E. coli* is ~0.8, it drops to ~0.25 with 31.25 µM AgF or AgNO_3_ + NaF. Similarly, with *S. epidermidis* MIC is 125 µM with AgNO_3_, with AgF and with AgNO_3_ + NaF, Figure 3C. However, the observation of the *S. epidermidis* surviving fractions in pre-biofilm conditions, shown in Figure 3D, evidences that at both 31.25 µM and 62.5 µM concentrations, the surviving fraction with AgNO_3_ is ~1.0, while it drops to ~0.30 (31.25 µM) and ~0.10 (62.5 µM) for both AgF and and AgNO_3_ + NaF. Finally, also in pre-biofilm conditions, the examination of the effect of the sole NaF on biofilms of *E. coli* and *S. epidermidis* do not show significant reduction of the surviving fraction (Supplementary Materials SM4). Interestingly, in pre-biofilm conditions the combination of AgNO_3_ + NaF gives lower surviving fractions than AgF, at a given concentration, for both *E. coli* (Figure 3B) and and *S. epidermidis* (Figure 3D). This prompted us to examine the surviving fraction of both strains when treated with NaNO_3_, both in pre-biofilm conditions and as planktonic bacteria, Supplementary Materials SM5. No surviving fraction decrease was observed in any case when concentration was 1000 µM or lower (i.e., at the NO_3_^−^ concentrations used in combination with AgF or for AgNO_3_). However, in pre-biofilm conditions, at NaNO_3_ concentrations >1000 µM (i.e., >>1 mM) a significant decrease of the surviving fraction was observed for *E. coli* and a smaller one for *S. epidermidis* RP62A (the effect on planktonic cells was instead negligible also in this higher concentration range). The results are in agreement with the studies of Schlag and coworkers on the effect of NO_3_^−^ on *S. epidermidis* RP62A [31], which revealed an adverse effect on biofilm formation at NO_3_^−^ concentrations >2 mM. The lower surviving fraction in pre-biofilm conditions observed for AgNO_3_ + NaF with respect to AgF at a given concentration (but <<1000 µM), can thus be attributed to the simultaneous action of Ag^+^, F^−^ and NO_3_^−^.

**Figure 4:**
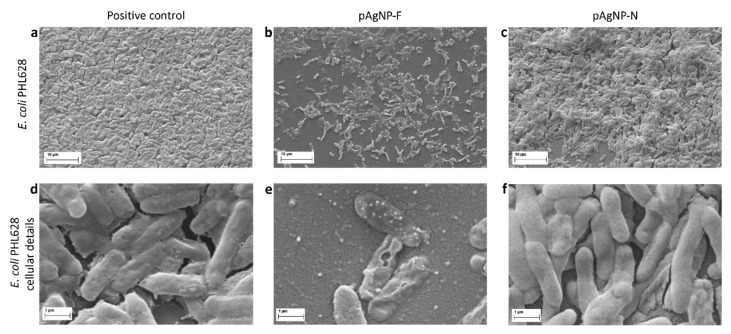
Scanning electron microscopy (SEM) imaging on *E. coli PH628*, treated with pAgNP-F and pAgNP-N in pre-biofilm conditions. (**A**): positive control (incubation in biofilm-inducing medium); (**B**): treatment with 250 µM pAgNP-F; (**C**): treatment with 250 µM pAgNP-N; (**D**–**F**): same as (**A**–**C**), respectively, at larger magnification.

Finally, to further support our observations on the synergic effect of AgNP and F^−^, we investigated by SEM (scanning electron microscopy) imaging the action of pAgNP-F with respect to pAgNP-N in pre-biofilm conditions. We have worked on *E. coli* at a 250 µM silver concentration. In these conditions (Figure 3B), while a significant surviving fraction of *E. coli* is found with pAgNP-N (~80%), the surviving fraction with pAgNP-F drops to zero (SEM analysis on *S. epidermidis* pre-biofilm treatment was deemed not informative since no substantial difference in viability is observed at any of the concentrations of pAgNP-F or p-AgNP-N used). Bacteria were seeded on a Thermanox^TM^ coverslips in medium containing 250 µM pAgNP-F or pAgNP-N and incubated for 24 h at 37 °C degrees. In Figure 4 we compare the positive control, which was only incubated in a biofilm-inducing medium, Figure 4A,D, with bacteria treated with 250 µM pAgNP-F, Figure 4B,E, and treated with 250 µM pAgNP-N, Figure 4C,F. 

Images at lower magnification (A-C) evidence a less well formed biofilm in the case of *E.coli* treated with pAgNP-N (Figure 4C) with respect to the control (Figure 4A). In the case of E. coli treated with pAgNP-F (Figure 4B) biofilm was indeed not formed. Moreover, observing images at higher magnification, while *E. coli* PHL628 cells appear intact both in the positive control (Figure 4D) and after treatment with pAgNP-N (Figure 4F), cells treated with pAgNP-F are instead clearly damaged. 

When post-biofilm conditions were taken into account, the higher resistance of the preformed biofilm unfortunately affects the MIC values exerting a levelling effect, see Supplementary Materials SM6. When nanoparticles were considered, we did not observed a reduction to 0 of the surviving fractions both for *E. coli* and *S. epidermidis*, although 500 µM pAgNP-F and pAgNP-N + NaF gave a significant surviving fraction reduction, similarly to what found for pAgNP-N [10]. Only by using Ag^+^, we were able to observe a MIC of 125–250 µM for either AgNO_3_ or AgF AgNO_3_ + NaF on *E. coli* biofilm.

## 3. Materials and Methods

### 3.1. Materials

Pectin from citrus peel (galacturonic acid ≥74%, dried basis), silver nitrate (≥99%), silver fluoride (≥99.9%), sodium citrate dihydrate (≥99%), sodium nitrate (≥99.0%), sodium hydroxide (≥98%), sodium borohydride (99%), ethanol (99.8%) were all purchased from Sigma-Aldrich (Milano, Italy) and used without further purification. Water was bidistilled from deionized water prior to use.

### 3.2. Synthesis of pAgNP-N and pAgNP-F 

The glassware used in the syntheses was washed with aqua regia and then three times with bidistilled water prior to use. Syntheses followed the protocol described in ref 10. Briefly, pectin from citrus peel was dissolved in 50 mL bidistilled water in 1% *w/w* concentration by stirring at 60 °C for 20 min. To this solution cooled at RT, 500 µL AgNO_3_ 0.1 M or 500 µL AgF 0.1 M were added to prepare pAgNP-N or pAgNP-F, respectively (Ag^+^ concentration was 0.001 M in all cases). Solutions were made 0.025 M in OH^–^ (pH range 10.5–11.0) by micro additions of 0.500 M standard NaOH. For initial characterization studies, pAgNP-F were obtained both by stirring such mixture for 24 h at RT, or for 4 h at 60 °C. The latter was used as the standard synthesis for the pAgNP-F solutions used for all bacteria treatments. The pAgNP-N solutions were also prepared by stirring for 4 h at 60 °C. The obtained yellow-brown colloidal solutions of pAgNP-N and pAgNP-F were kept in a stoppered flask at RT and used with no other treatment.

Only for carrying out TGA and FT-IR experiments, we prepared dry pAgNP-F samples. These were obtained by adding 1 mL of pAgNP-F colloidal solution to 9.0 mL of absolute ethanol. The obtained turbid mixture was then ultracentrifuged at 13,000 rpm (15870 *g*) for 45 min, obtaining a pellet at the bottom that was separated from the supernatant, dried with a N_2_ flux for 1 h, then in a vacuum desiccator for 14 h.

### 3.3. Methods

*FT-IR spectra*. Such measurements were carried out on the various samples as dry powders, using the attenuated total reflectance mode. The instrument was a NICOLET iS10 (Nicolet, Madison, WI, USA). 256 scans were carried out, at a resolution of 4 cm^–1^ and data spacing of 0.482 cm^–1^ in the 4000–650 cm^−1^ range. A collected background (FT-IR for empty sample holder) was measured for each sample, thus making spectral subtraction under the same sample conditions.

*TGA analysis*. About 8 mg of the same dry samples were analyzed by thermogravimetry in a Q5000 Instrument (TA Instruments Inc, New Castle, DE, USA) by heating into a Pt pan from 25 °C up to 1000 °C (10 K/min, nitrogen flow 100 mL/min).

*Free Ag^+^ determination.* A potentiometric titration was used to obtain the Ag^+^ content in the pAgNP-F solutions, by means of the standard addition method [25]. A combined silver electrode (silver bar, d = 0.8 cm) and a Hg/Hg_2_SO_4_ (saturated K_2_SO_4_) were used (Radiometer Analytical, MC6091Ag-9). The potentiometer was a Akralab Nahita 903 (precision: ±0.1 mV; Alicante, Spain). The supporting electrolyte was KNO_3_, added in 0.1 M concentration. A linear calibration curve was obtained by adding known amounts of AgF (from 0.1 up to 200 mg/L) to a 1% *w/w* pectin solution (0.1 M in KNO_3_) and plotting log [Ag^+^] vs E (mV). The obtained straight line has a Nernstian slope (ca 60 mV for ten-fold change in concentration). Experiments were carried out at 20 °C in less than 0.5 h, in the absence of added base. No silver reduction by pectin took place under these conditions.

*TEM imaging*. TEM images were taken on a Jeol JEM-1200 EX II instrument (Jeol Italia SPA, Basiglio, Italy) on 10 μL samples, deposited on Nickel grids (300 mesh) covered with a Parlodion membrane

*HRTEM imaging*. High Resolution TEM imaging of the samples was performed with a FEI Titan Cubed microscope (Thermo Fischer Scientific, Eindhoven, Netherlands), working at an acceleration voltage of 300 kV and equipped with a ultra-bright Field Emission Gun (X-FEG), and a spherical aberration (C_s_) Image corrector, with an ultimate point resolution of 0.09 nm. The images were acquired and recorded by using a Gatan 2kX2k CCD camera (Gatan, Pleasanton, CA, USA).

### 3.4. Bacterial Strains, Culture Condition and Evaluation of Antimicrobial Activity of AgNPs and Related Salts 

Microorganisms *Escherichia coli* PHL628 and *Staphylococcus epidermidis* RP62A [PMID: 3679536] are biofilm-producing strains, kind gifts from Prof. Dr. Roberta Migliavacca (University of Pavia, Pavia, Italy) and Prof. Timothy J. Foster (Department of Microbiology, Dublin University, Dublin, Ireland), respectively. An overnight culture of *E. coli* PHL628 or *S. epidermidis* RP62A was grown in Luria Bertani Broth (LB) and in tryptic soy broth (TSB) (Difco Laboratories Inc., Detroit, MI, USA), respectively, under aerobic conditions at a temperature of 37 °C using a shaker incubator (Certomat^TM^ BS-T, B.Braun Biotech International, Melsungen, Germany) at 200 rpm.

All soluble salts (AgNO_3,_ AgF, NaF) were filter-sterilized (0.22 µm) prior antibacterial activity tests; p-AgNPs sample preparation and bacterial experiments were performed essentially as reported in our previous work [10].

*Planktonic cell conditions*. Briefly, Minimum Inhibitory Concentration (MIC) values were determined for pAgNP-N, p-AgNP-F and respective Ag salts, and their combinations. Overnight-grown bacteria were diluted in appropriate fresh medium to 1 × 10^4^ cells/mL. Dilution of indicated AgNPs suspensions or salts were titrated starting from the initial concentration of 1 mM for successive 11 steps in 100 µL (1:2 dilution ratio). Then, 100 µL of the diluted bacterial suspension was added to the microtiter plate and incubated for 24 h at 37 °C in static conditions. Cell viability was estimated through the quantitative 3-[4,5-dimethylthiazol-2-yl]-2,5diphenyltetrazoliumbromide (MTT). MTT, dissolved in PBS (0.134 M NaCl, 8.34 mM Na_2_HPO_4_, 1.64 mM NaH_2_PO_4_, pH 7.4), was prepared at 5 mg/mL (stock solution) and diluted at 0.5 mg/mL for the viability tests. Reduction of the MTT salt, mediated by cellular respiration, results in purple insoluble formazan granules that are dissolved through acidified 2-propanol (0.04 M HCl). Spectrophotometric record of the results was performed with Clariostar^TM^ microplate reader (BMG-Labtech, Ortenberg, Germany) at 570 nm (subtracted reference wavelength was 630 nm). Surviving fraction was calculated by dividing the number of bacteria survived in presence of AgNPs or AgNO_3_ by number of bacteria grown in absence of any treatment.

*Biofilm culture conditions*. To induce biofilm formation, the starter cultures were diluted in 0.25% glucose-containing medium to induce biofilm formation. *E. coli* PHL628 was diluted at a final ratio of 1:100 in LB medium and *S. epidermidis* RP62A at 1:200 in TSB (both media supplemented with glucose). Pre-biofilm conditions: geometric dilutions of p-AgNPs or salts in glucose-enriched medium were incubated with 100 µL of bacterial suspension for 24 h as previously described. Post-biofilm conditions: starter cultures were diluted in corresponding glucose-supplemented medium and bacteria were seeded to form biofilm for 24 h at 37 °C. The supernatant, containing planktonic cells, was carefully removed and AgNPs suspensions or salts already diluted in the appropriate glucose-containing medium were added to the formed biofilm and incubated for further 24 h.

In both culture conditions, at the end of the incubation period, biofilm was vigorously disrupted. The viability was assessed through MTT assay as described above. Experiments were performed in triplicate, at least twice.

### 3.5. SEM Imaging of E.coli in Pre-Biofilm Condition

Bacteria were cultured and diluted as reported above and seeded on a Thermanox^TM^ coverslips (Nunc) in 1 mL final volume containing 250 μM p-AgNP in 24-well cell culture plate. Bacteria in the positive control were incubated with medium only. After 24 h at 37 °C, medium was carefully removed and samples were fixed with 2.5% (*v/v*) glutaraldehyde in 0.1 M Na-cacodylate buffer, pH 7.2, for 1 h at 4 °C. After additional washes with cacodylate buffer to remove the excess of glutaraldehyde, the samples were dehydrated with two washes of 10 min in 96% ethanol. After freezing, the samples were then lyophilized for 3 h using an Emitech (Ashford, UK) K- 850 apparatus and placed on a mounting base. Finally, coverslips were fixed on aluminum stubs with a C bi-adhesive tape and sputtered with gold (3 times for 1 min at 10 mA). Analyses were carried out using a Zeiss EVO-MA10 scanning electron microscope (Carl Zeiss Microscopy GmbH, Oberkochen, Germany), acceleration voltage used was 20 kV, working distance 8,5 mm. Scale bar was inserted automatically by the instrument. 

## 4. Conclusions

AgNP can be prepared with pectin both from AgF or AgNO_3_, obtaining identical products on the chemical-physical point of view. However, the data collected on planktonic strains and in pre-biofilm treatment conditions show a synergic effect between AgNP and the F^−^ anion on both *E. coli* and *S. epidermidis*. A similar synergic effect is also observed between the Ag^+^ ion and F^−^ when using silver salts instead of nanoparticles. Noticeably, this effect holds even at F^−^ concentrations three orders of magnitude lower than the 200 mM concentration reported by Baker and coworkers [21] for the MIC of NaF on an unmodified *E. coli* strain. In the attempt to find an explanation to this unexpected phenomenon, it should be noticed that Baker and coworkers also demonstrated that the F^−^ anion can be toxic even at micromolar concentrations if the *E. coli* strain has been modified with a genetic knockout (KO) of its crcB gene [21]. The perusal of related literature also allows us to understand that the crcB gene is associated with a fluoride-responsive riboswitch [21,32] that drives the expression of CrcB transport proteins having the role of reducing fluoride concentrations in *E. coli* cells, thus strongly mitigating the anion toxic effect. In this regard, it is relevant to stress that silver (I) ions [33,34] and AgNP [35] are known to strongly interact with RNA and bacterial membrane proteins [36,37]. We have no possibilities to experimentally study the interactions of Ag^+^ and AgNP with the crcB gene or the CrcB protein functions of the *E. coli* strain; however, we can at least put forward the hypothesis that Ag^+^ and AgNP might inhibit either the crcB gene or the CrcB protein functions, preventing the elimination of F^−^ from the bacterial cells. This could allow the F^−^ anion to exert a toxic effect even at micromolar concentrations. Finally, we also observed a small but clear effect of additional vitality reduction when AgNO_3_ and NaF are used as salts instead of AgF in pre-biofilm conditions. This could be connected to the additional inhibitory action on biofilm formation brought by the nitrate anion, which has been reported at two orders of magnitude larger concentrations than in our case. 

## Figures and Tables

**Table 1:** Chemical-physical data for pAgNP-F and pAgNP-N.

	d (nm)^a^	λ_max_ (nm)	free Ag^+^(ppm)^d^	% mass (pectin)	pH^d^
pAgNP-F	7.2(2.7)	412	0.75	34.5	10.6–11.2
pAgNP-N	7.8(2.0)^b^ 8.0 (2.6)^c^	412^b^ 412^c^	0.78^b^ 0.79^c^	38.0^b^ 37.0^c^	10.5–10.9^b^ 10.5–11.0^c^

^a^standard deviation in parenthesis; ^b^this work; ^c^Ref. 10; ^d^measured at the end of the synthesis.

## Supplementary Materials

The following are available online. SM1: TEM images of pAgNP-F obtained at 25 and 60 °C, HRTEM image of pAgNP-N; SM2: FTIR spectra of pAgNP-F and pectin; SM3: thermogravimetric analysis of pAgNP-F; SM4: surviving fractions (E. coli and S. epidermidis) on NaF treatment, in pre-biofilm conditions; SM5: surviving fractions on treatment of *E. coli* and *S. epidermidis* with NaNO_3_; SM6: surviving fraction in post-biofilm treatments
