# Supplementary material for: Increased Antibacterial and Antibiofilm Properties of Silver Nanoparticles Using Silver Fluoride as Precursor
Source: Molecules. 2020 Jul 31;25(15):3494. doi: 10.3390/molecules25153494 (PMC7436145; doi:10.3390/molecules25153494)
Supplement: Supplementary file 1 [file molecules-25-03494-s001.pdf]

Increased antibacterial and antibiofilm properties of silver nanoparticles using silver fluoride as precursor

Federico Bertoglio,* Lorenzo De Vita, Agnese D'Agostino, Yuri Diaz Fernandez, Andrea Falqui, Alberto Casu, Daniele Merli, Chiara Milanese, Silvia Rossi, Angelo Taglietti, Livia Visai, Piersandro Pallavicini,*

SUPPLEMENTARY MATERIALS

SM1. TEM and HRTEM imaging

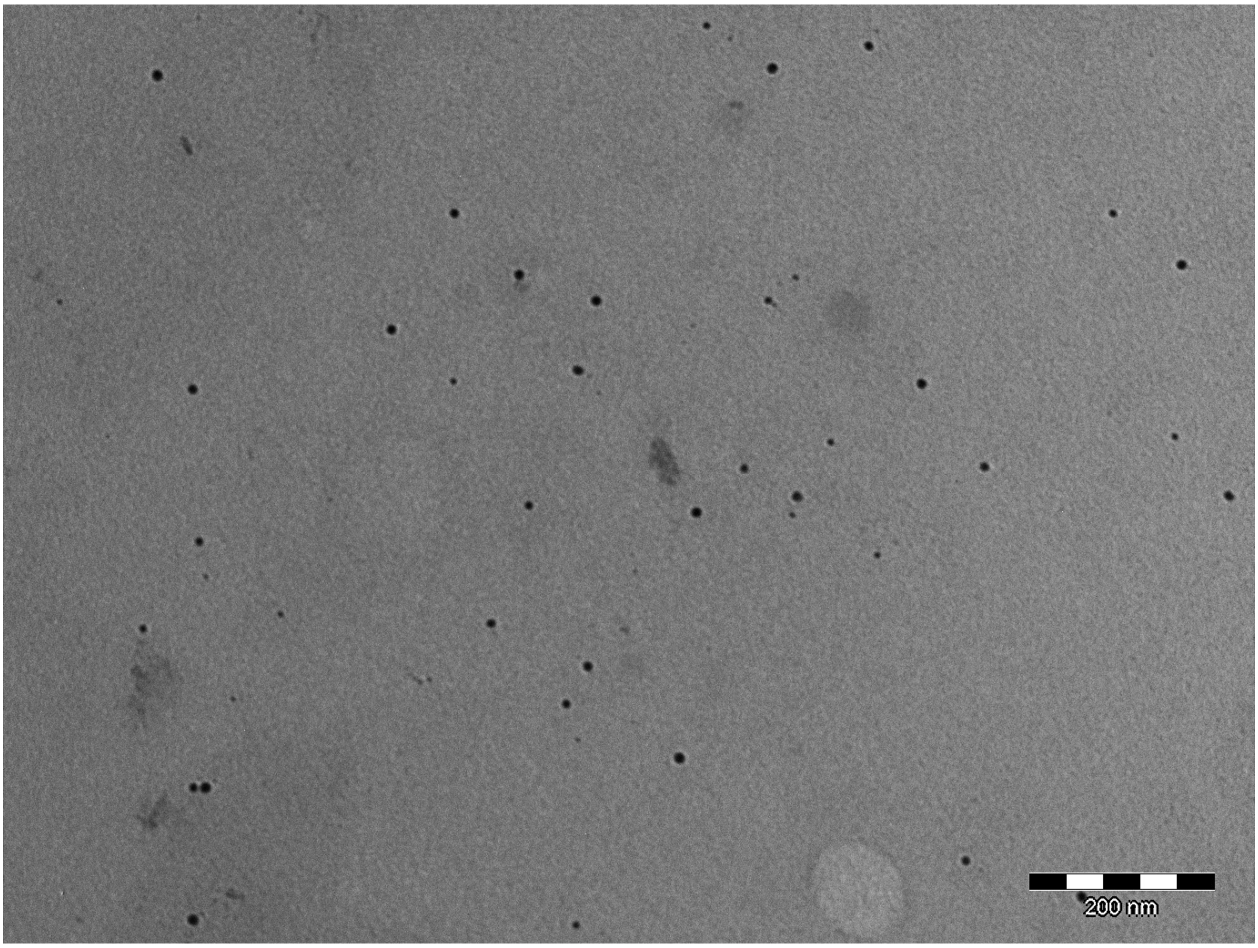

SM1.1 Large TEM image of pAgNP-F obtained at 25 °C

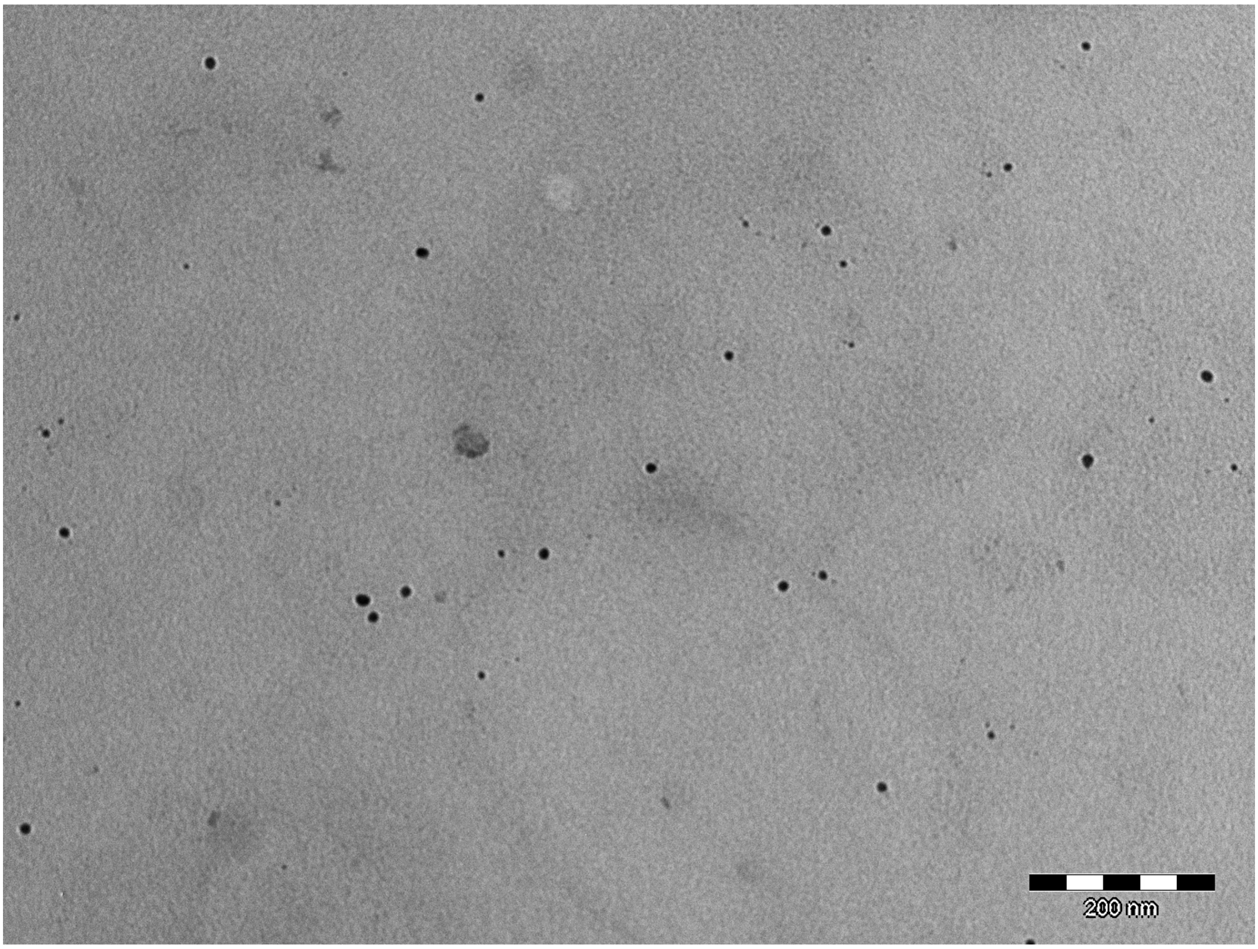

SM1.2 Large TEM image of pAgNP-F obtained at 60 °C

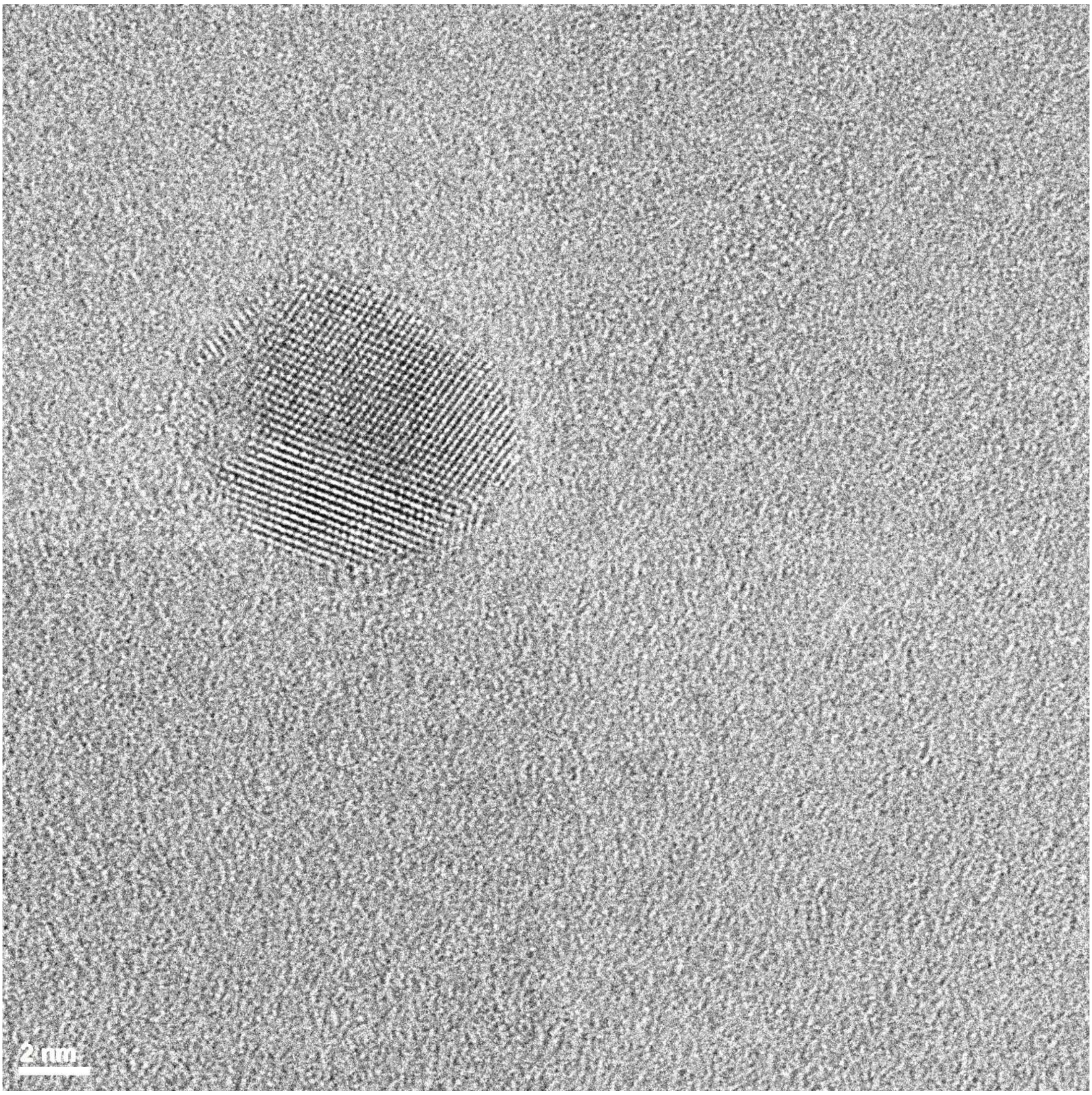

SM1.3 HRTEM image of pAgNP-N, obtained at 60 °C

SM2. FTIR spectrum of pAgNP-F

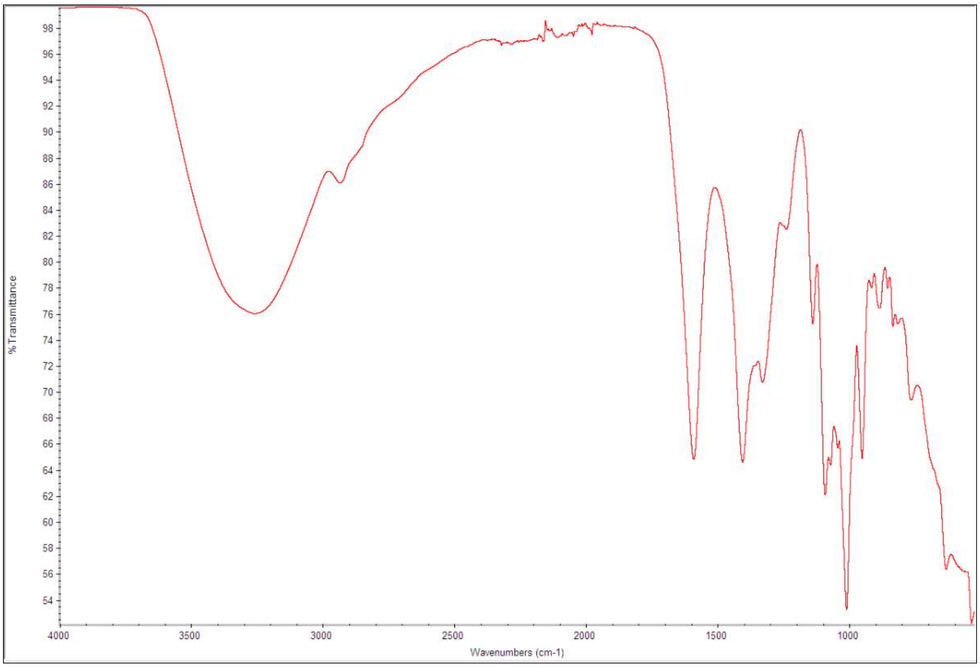

SM2.1 FTIR spectrum of pAgNP-F (obtained by synthesis at 60 °C)

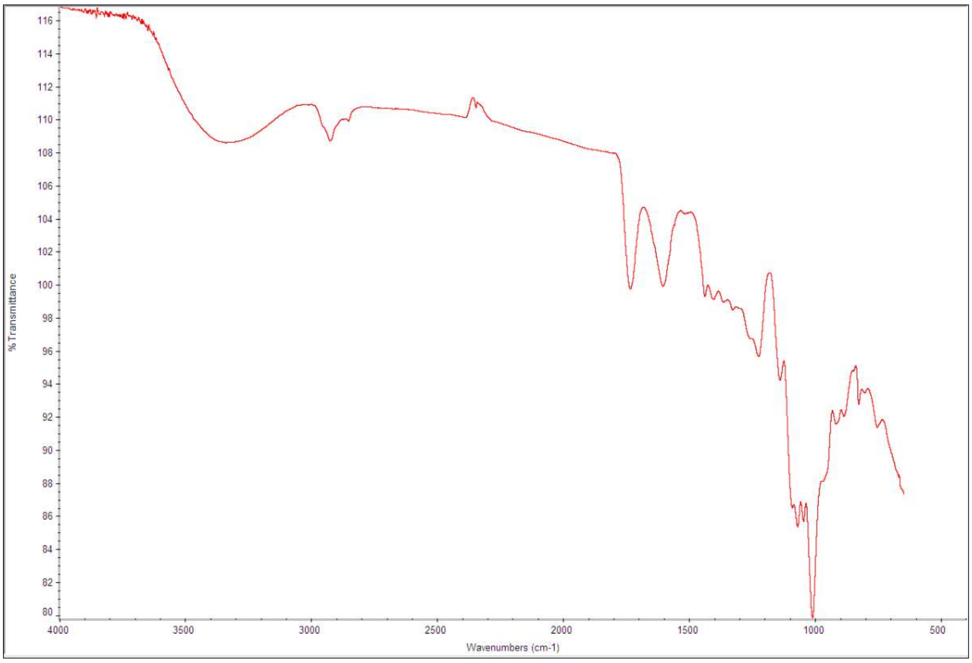

SM2.2 FTIR spectrum on pure pectin from citrus

SM3. Thermogravimetric Analysis

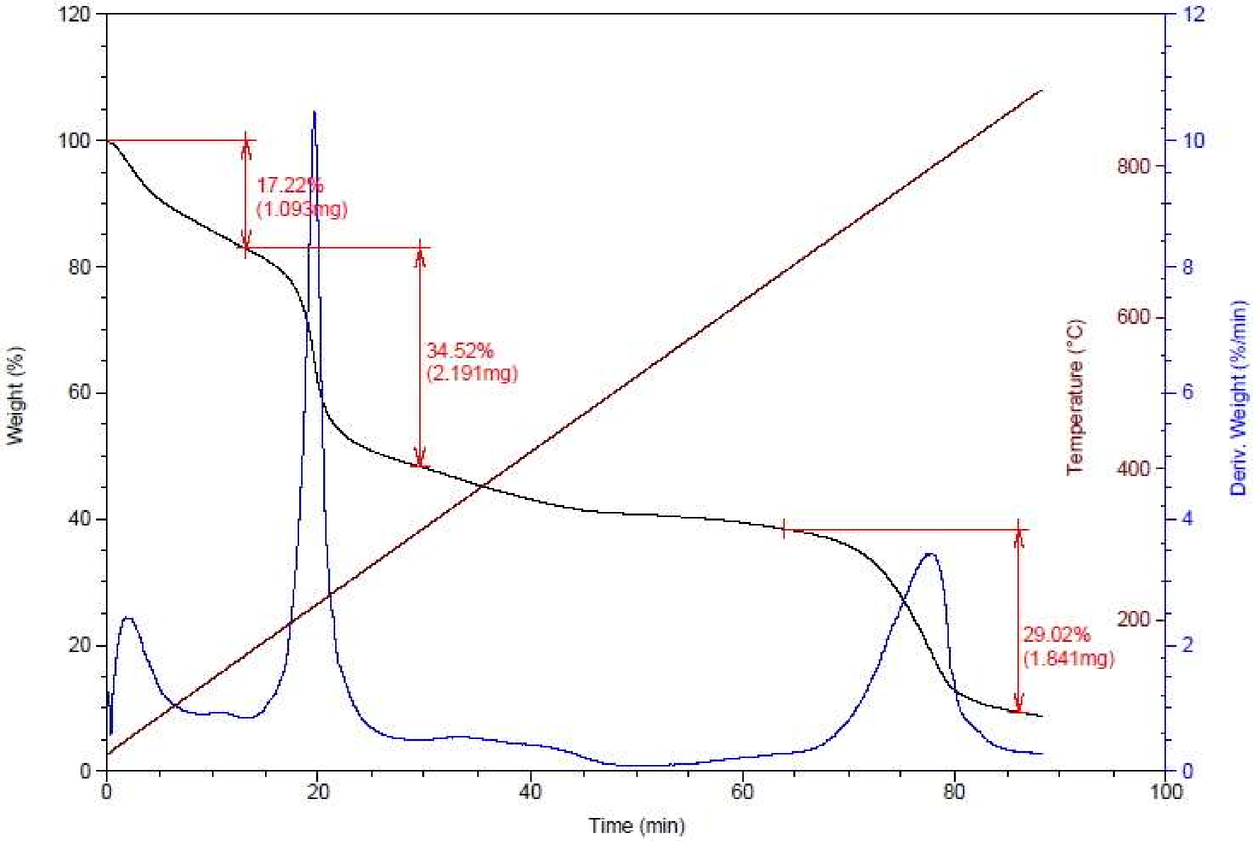

SM3.1. Thermogravimetric analysis on solid isolated pAgNP-F (synthesis at 60 °C). Temperature ramping (straight line, right vertical axis, black scale) 20-900 °C

SM4 – Surviving fractions on NaF treatment (pre-biofilm conditions)

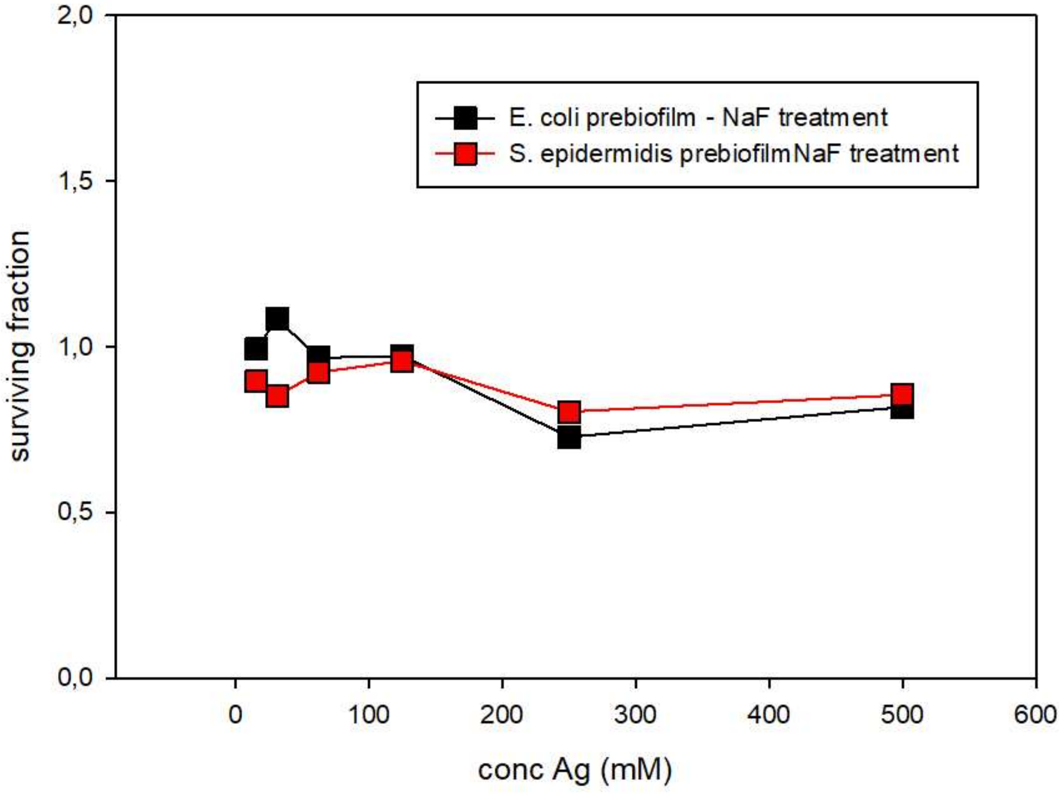

SM4.1 Surviving fractions of *E. coli* (black squares) and *S. epidermidis* (red squares) on treatment with NaF in pre-biofilm conditions

SM5. Surviving fractions on treatment of *E. coli* and *S. epidermidis* with with $NaNO_3$ (all conditions)

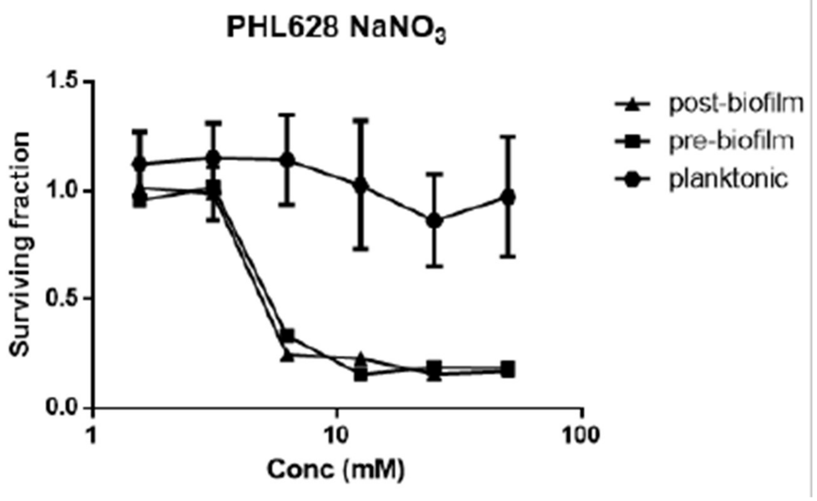

SM5.1 *E. coli PHL628* treatment with $NaNO_3$, all conditions (please note that the scale is in millimoles and not in micromoles as in the experiments with silver)

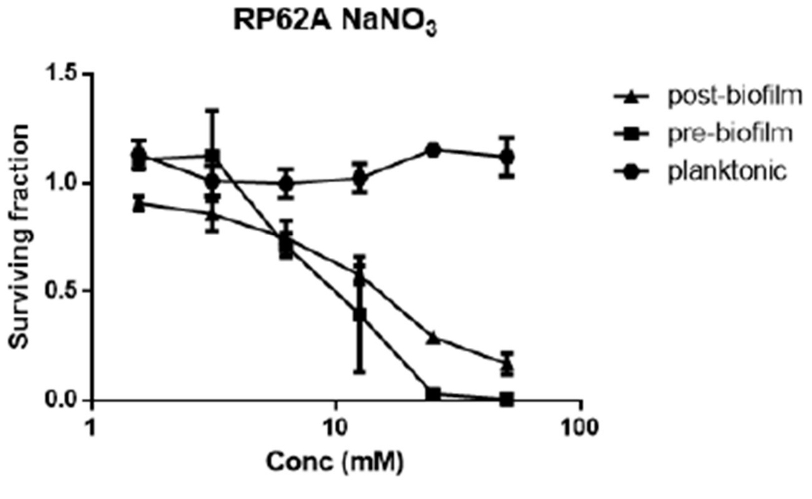

SM5.2 *S. epidermidis RP62A* treatment with $NaNO_3$, all conditions (please note that the scale is in millimoles and not in micromoles as in the experiments with silver)

SM6. Post-biofilm treatments

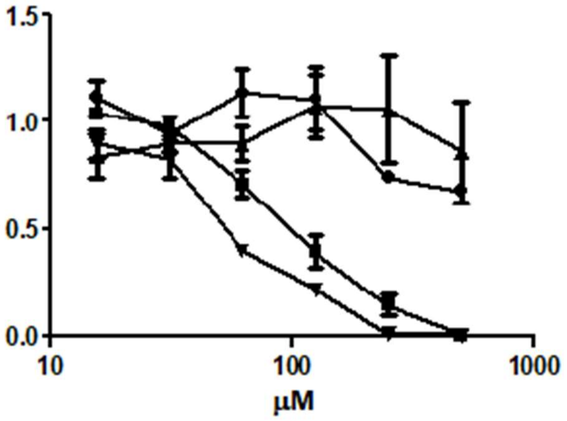

SM6.1. Surviving fraction of *E. coli PHL628* in post-biofilm conditions. Treatment with pAgNP-F (circles), pAgNP-N + NaF (triangles up), AgF (squares) and $AgNO_3$ + NaF (triangles down)

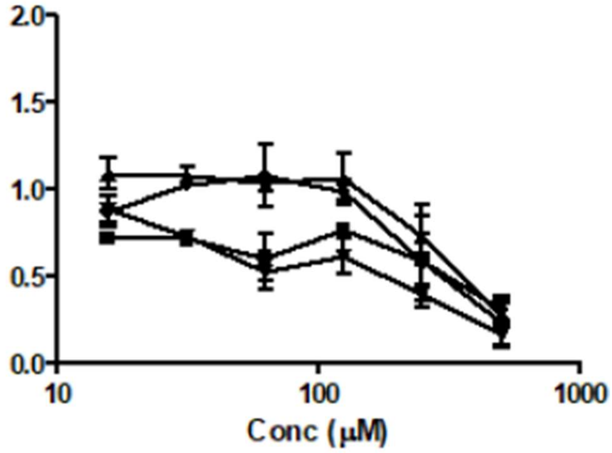

SM6.2 Surviving fraction of *S. epidermidis RP62A* in post biofilm conditions. Treatment with pAgNP-F (circles), pAgNP-N + NaF (triangles up), AgF (squares), $AgNO_3$ + NaF (triangles down)
